# An Invasive Fish and the Time-Lagged Spread of Its Parasite across the Hawaiian Archipelago

**DOI:** 10.1371/journal.pone.0056940

**Published:** 2013-02-27

**Authors:** Michelle R. Gaither, Greta Aeby, Matthias Vignon, Yu-ichiro Meguro, Mark Rigby, Christina Runyon, Robert J. Toonen, Chelsea L. Wood, Brian W. Bowen

**Affiliations:** 1 Ichthyology, California Academy of Sciences, San Francisco, California, United States of America; 2 Hawai'i Institute of Marine Biology, University of Hawai'i at Mānoa, Kane'ohe, Hawai'i, United States of America; 3 UMR 1224 Ecobiop, UFR Sciences et Techniques Côte Basque, Univ Pau and Pays Adour, Anglet, France; 4 UMR 1224 Ecobiop, Aquapôle, INRA, St Pée sur Nivelle, France; 5 Division of Marine Biosciences, Graduate School of Fisheries Sciences, Hokkaido University, Hakodate, Japan; 6 Parsons, South Jordan, Utah, United States of America; 7 Marine Science Institute, University of California Santa Barbara, Santa Barbara, California, United States of America; 8 Department of Biology, Stanford University, Stanford, California, United States of America; 9 Hopkins Marine Station, Stanford University, Pacific Grove, California, United States of America; Leibniz Center for Tropical Marine Ecology, Germany

## Abstract

Efforts to limit the impact of invasive species are frustrated by the cryptogenic status of a large proportion of those species. Half a century ago, the state of Hawai'i introduced the Bluestripe Snapper, *Lutjanus kasmira*, to O'ahu for fisheries enhancement. Today, this species shares an intestinal nematode parasite, *Spirocamallanus istiblenni*, with native Hawaiian fishes, raising the possibility that the introduced fish carried a parasite that has since spread to naïve local hosts. Here, we employ a multidisciplinary approach, combining molecular, historical, and ecological data to confirm the alien status of *S. istiblenni* in Hawai'i. Using molecular sequence data we show that *S. istiblenni* from Hawai'i are genetically affiliated with source populations in French Polynesia, and not parasites at a geographically intermediate location in the Line Islands. *S. istiblenni* from Hawai'i are a genetic subset of the more diverse source populations, indicating a bottleneck at introduction. Ecological surveys indicate that the parasite has found suitable intermediate hosts in Hawai'i, which are required for the completion of its life cycle, and that the parasite is twice as prevalent in Hawaiian Bluestripe Snappers as in source populations. While the introduced snapper has spread across the entire 2600 km archipelago to Kure Atoll, the introduced parasite has spread only half that distance. However, the parasite faces no apparent impediments to invading the entire archipelago, with unknown implications for naïve indigenous Hawaiian fishes and the protected Papahānaumokuākea Marine National Monument.

## Introduction

The rate of species introductions has increased dramatically in modern times, correlating with human population growth, advances in transportation, and increased international trade [1], [2]. While most introduced species never become established, those that persist can have serious economic impacts [3], [4], consequences for human health [5], and can pose a significant threat to biodiversity and ecosystem function [6]–[8]. In response to these risks, resource managers and government agencies are dedicated to the identification, control, and eradication of non-indigenous species (NIS) [9]–[11].

Efforts to stem the impact of invasive species are impeded by the uncertain or cryptogenic status of many NIS [12], [13]. For example, in the San Francisco Bay an estimated 37% of known or suspected alien species are cryptogenic [12]. These species leave resource managers with an uncertain course of action and are a potential drain on limited management resources. Identifying the native range of cryptogenic species is hampered by the paucity of fossil and historical records, and is particularly problematic among parasites and microbes whose taxonomies are poorly resolved relative to those of more prominent plants and animals [14].

In the absence of natural range data or fossil records, a multi-disciplinary approach combining phylogeography, population genetics, and ecology may illuminate the status of cryptogenic species. Here, we employ such an approach to resolve the status of the parasitic nematode *Spirocamallanus istiblenni* (Noble 1966, family Camallanidae), which may have been introduced to Hawai'i during well-intentioned fish introductions.

**Figure 1:**
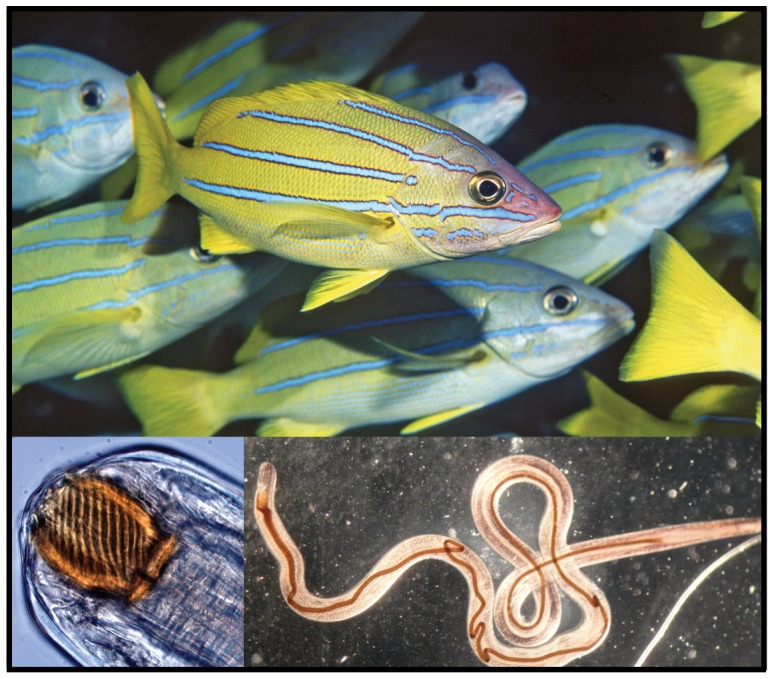
The introduced Bluestripe Snapper *Lutjanus kasmira* and the parasitic nematode *Spirocamallanus istiblenni*. Photo credits: Greta Aeby, Keoki Stender, and Chelsea Wood.

**Figure 2:**
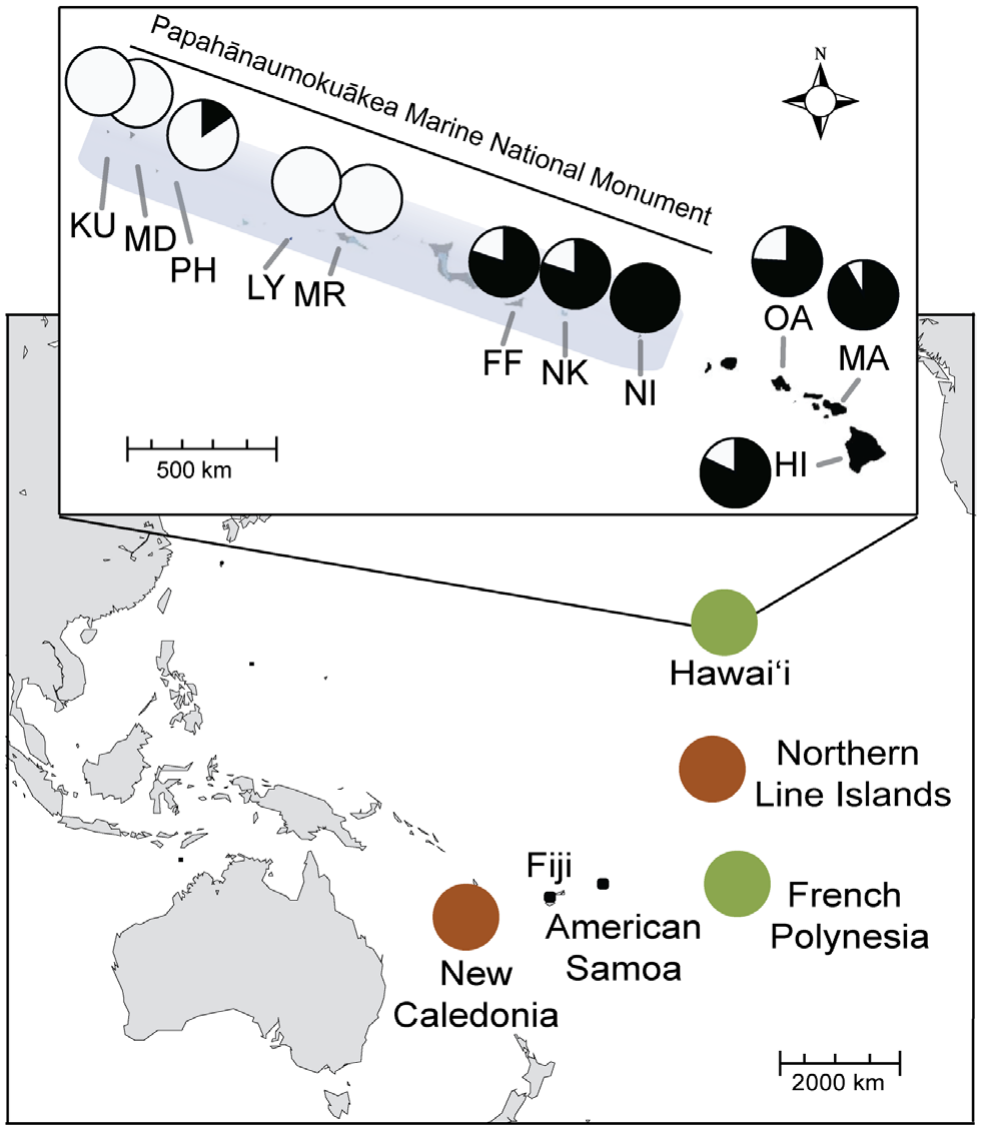
Map of sample locations for three species of host fish. Abbreviations for sample locations, species, and number of specimens collected are listed in Table 1. Colored circles delineate the two divergent lineages detected in phylogenetic analyses (Fig. 3). Pie charts for Hawai'i locations represent prevalence data for the host fish *Lutjanus kasmira* (black = proportion infected; white = proportion not infected). Main Hawaiian Islands listed include HI, MA, OA, and KA.

In an effort to enhance local fisheries, the Hawai'i Division of Fish and Game transplanted the Bluestripe Snapper *Lutjanus kasmira* (Forsskål 1775, family Lutjanidae; Fig. 1) to the island of O'ahu, including 2435 fish from the Marquesas Islands in 1958 and 728 fish from the Society Islands in 1961 (Fig. 2) [15], [16]. (Note: The Marquesas and Society Islands are two of the four primary archipelagos in French Polynesia.) Prior to release, the fish were treated with copper sulfate to remove external parasites [17], [18]. No measures were taken to eliminate internal parasites. Following introduction, *L. kasmira* spread rapidly, reaching the far northwestern end of the archipelago, over 2000 km from the introduction site, within 34 years (Fig. 2).

Despite measures taken to prevent the introduction of parasites, faunal comparisons between Hawai'i and French Polynesia indicate that up to eight species of ectoparasitic flatworms (class Monogenea) were introduced to Hawai'i, plus two cryptogenic species, including the endoparasitic nematode *S. istiblenni* (Fig. 1) [18], [19]. Camallanids attach to the lining of the gastro-intestinal tract where they feed on host blood and tissue. At high densities, these parasites can cause severe damage to intestinal tissues [20], [21]. In Hawai'i, *S. istiblenni* is known to parasitize *L. kasmira* and at least seven native species of fish (Text S1). The parasite has been documented throughout the Main Hawaiian Islands (near the point of fish introduction) and at high prevalence as far west as French Frigate Shoals in the middle of the archipelago (Fig. 2). While considerable effort has been invested in documenting the range (and possible spread) of this parasite, its alien status remains uncertain.

In general, the geographic distribution of fish parasites in the Pacific is poorly documented, and the uncertainty in the natural range of *S. istiblenni* stems from a lack of occurrence data. Outside of Hawai'i, *S. istiblenni* is known only from French Polynesia and Fiji [22], [23] (reexamination of materials from Okinawa has called into question the accuracy of earlier records [23]). Based on biogeographic data from reef fish species, the most likely route of natural dispersal between the South Pacific and Hawai'i is along the Line Islands, which straddle the equator 1,400 km south of Hawai'i. The presence of several species (or genetic lineages) in the Line Islands and Hawai'i, but not elsewhere in the Pacific, confirms this avenue of dispersal [24], [25]. Therefore, natural colonization of the Hawaiian Islands by this parasite remains a possibility.

Here, we describe a multidisciplinary approach, combining phylogeography and population genetics with ecological survey data from the native and introduced ranges, to resolve the cryptogenic status of *S. istiblenni* in Hawai'i. Specifically, we surveyed host fish from across much of the known range of *S. istiblenni* to answer the following questions: 1) Is *S. istiblenni* found in the Line Islands, the closest archipelago to Hawai'i and a known gateway for natural dispersal into the Hawaiian Islands? 2) Are *S. istiblenni* in Hawai'i genetically divergent from other populations in the Pacific and, therefore, likely to be native to those islands, or 3) Do *S. istiblenni* in Hawai'i share a genetic affinity with French Polynesia (the native range of the host fish *L. kasmira*), indicating a likely human-mediated introduction? This study also benefits from robust historical records on the fish introduction, a rare advantage in studies of marine invasions.

## Methods

### Specimen collection and dissection

This study was carried out in strict accordance with recommendations in the Guide for the Care and Use of Laboratory Animals of the National Institutes of Health. The protocol was approved by the Committee on the Ethics of Animal Experiments of the University of Hawai'i (Permit Number: 09746). To assess the prevalence of *S. istiblenni* in Hawai'i, a total of 288 specimens of the host fish *L. kasmira* were collected from 11 locations across the archipelago by scuba divers using polespears (State of Hawai'i Division of Aquatic Resources Special Activity Permits SAP-2008-99 & SAP-2009-101; Table 1, Fig. 2). All fish were pithed immediately after collection, as required under permit. Specimens from the uninhabited Northwestern Hawaiian Islands were obtained during research expeditions on the NOAA R/V Hi'ialakai, as part of an initiative by the Papahānaumokuākea Marine National Monument (http://hawaiireef.noaa.gov/) to monitor and characterize this vast protected area (National Oceanographic and Atmospheric Administration permits PMNM-2008-046 & PMNM-2009-044). To determine whether *S. istiblenni* in Hawai'i are of French Polynesian origin, we conducted surveys of several known host species at locations across the Central Pacific (Table 1). Forty *L. kasmira* were collected from Fiji (Fig. 2). Our collection efforts in the Line Islands were divided between Kiritimati and Palmyra (1,800 km and 1,500 km south of Hawai'i, respectively). Due to logistic constraints and a scarcity of *L. kasmira* in parts of the Line Islands, we collected only three at Kiritimati and none at Palmyra. However, we were able to obtain two other *S. istiblenni* hosts, the Blacktail Snapper *L. fulvus* (N = 131) and the Peacock Grouper *C. argus* (N = 199). Parasitic nematodes were recovered from intestinal tissue and preserved in either 95% ethanol (EtOH) or saturated NaCl solution [26], and stored at room temperature. Nematodes were visually identified to at least the level of genus (*Spirocamallanus*) while the species designation was confirmed for a subset of the Hawaiian specimens. A subset of the *S. istiblenni* collected by Vignon et al. [18] from *L. kasmira* in French Polynesia, as well as specimens collected during a field expedition to the region in 2010, were used for genetic analyses. In total, *S. istiblenni* from 32 *L. kasmira* from the Marquesas and 10 *L. kasmira* from the Society Islands were utilized for a total of 119 parasites (Table 2). Voucher specimens were deposited in the National Museum of Natural History, Paris, France (Table S1).

**Table 1:** Summary statistics for spirocamallanids collected from three host species.

	*N*	Prevalence (%)	Intensity	Range
***Lutjanus kasmira***				
French Polynesia				
Marquesas Islands (MI)	72	52.7	4.0±0.4	1–12
Society Islands (SI)	231	29.0	1.7±0.1	1–8
All French Polynesia	303	34.7	2.6±0.2	1–12
Hawai'i				
Hawai'i Island (HI)	28	78.6	3.3±0.4	1–9
Maui (MA)	49	91.8	7.5±0.9	1–22
O'ahu (OA)	68	76.5	7.1±0.7	1–22
Nihoa (NI)	11	100.0	9.5±2.1	2–25
Necker (NE)	24	79.2	6.7±1.3	1–22
French Frigate Shoals (FF)	40	80.0	7.3±1.1	1–24
Maro (MR)	1	0	–	
Laysan (LA)	8	0	–	
Pearl & Hermes (PH)	13	15.4	6.5±0.5	6–7
Midway (MD)	40	0	–	
Kure (KU)	6	0	–	
All Hawai'i	288	63.5	6.9±0.4	1–25
Northern Line Islands	7	0	–	
Fiji	40	7.5	1.3±0.3	1–2
American Samoa	14	0	–	
***Lutjanus fulvus***				
Northern Line Islands	131	0	–	
***Cephalopholis argus***				
Northern Line Islands	199	4.5	1.8±0.2	1–3

Sample location, number of hosts dissected (*N*), number of infected fish (*N*_I_), percent of fish that harbored parasites (prevalence), and the mean number (intensity ± standard error) and range of parasites per infected fish are listed. Northern Line Islands = Kiritimati and Palmyra. Data for French Polynesia are from Vignon et al. [18].

**Table 2:** Molecular diversity indices for COI sequences from *Spirocamallanus istiblenni*.

	*N*	*N_h_*	*h*	*π*
**French Polynesia**				
Marquesas Islands	70	16	0.73±0.05	0.005±0.003
Society Islands	49	4	0.26±0.08	0.001±0.001
All French Polynesia	119	18	0.58±0.05	0.003±0.002
**Hawai'i**				
Hawai'i Island	31	5	0.62±0.07	0.002±0.002
Maui	45	6	0.72±0.04	0.003±0.002
O'ahu	46	4	0.56±0.04	0.002±0.002
Nihoa	55	5	0.67±0.40	0.003±0.002
Necker	2	1	–	–
French Frigate Shoals	55	4	0.68±0.03	0.002±0.002
Maro	–	–	–	–
Laysan	–	–	–	–
Pearl & Hermes	6	3	0.73±0.16	0.003±0.002
Midway Atoll	–	–	–	–
Kure Atoll	–	–	–	–
All Hawai'i	240	7	0.67±0.02	0.003±0.002

Specimens were collected from the host fish *Lutjanus kasmira*. Number of specimens (*N*), number of haplotypes (*N*_h_), haplotype diversity (*h*), nucleotide diversity (*π*) as reported by arlequin 3.5 [36] are listed.

### DNA extraction, PCR amplifications, and sequencing

DNA was isolated using either an E.Z.N.A® Tissue DNA Kit (Omega Bio-Tek, Inc., Norcross, GA) following the manufacturer's protocol or the modified HotSHOT method [27], [28]. All genomic DNA was stored at −20°C. Approximately 420 bp of mitochondrial cytochrome oxidase I gene (COI) were amplified in all specimens using the primers FCOX1A and RCOX1A of Wu et al. [29]. A subset of these specimens was utilized for phylogenetic analyses. In these samples, two overlapping fragments of the ribosomal small subunit 18S (18S) were amplified using the primer pairs G18S4/647 and 652/647 of Nadler et al. [30] and approximately 175 bp of the ATP Synthetase Subunit β (ATPSβ) intron was amplified using the ATPSβf1 and ATPSβr1 primers of Jarman et al. [31].

Polymerase chain reactions (PCRs) for all three markers were carried out in a 10 µl volume containing 2–15 ng of template DNA, 0.2–0.3 µM of each primer, 5 µl of the premixed PCR solution BioMix Red™ (Bioline Inc., Springfield, NJ, USA), and deionized water to volume. PCR reactions utilized the following cycling parameters: initial denaturation at 95°C and final extension at 72°C (10 min each), with an intervening 35 cycles of 30 s at 94°C, 30 s at the annealing temperature (COI, 54°C; 18S, 58°C; ATPSβ, 58°C), and 45 s at 72°C. Amplification products were purified using 0.75 units of Exonuclease I: 0.5 units of Shrimp Alkaline Phosphatase (ExoSAP; USB, Cleveland, OH, USA) per 7.5 µl PCR products at 37°C for 60 min, followed by deactivation at 85°C for 15 min. DNA sequencing was performed with fluorescently-labeled dideoxy terminators on an ABI 3730XL Genetic Analyzer (Applied Biosystems, Foster City, CA, USA) at the University of Hawai'i Advanced Studies of Genomics, Proteomics and Bioinformatics sequencing facility.

Sequences for each locus were aligned, edited, and trimmed to a common length using the DNA sequence assembly and analysis software Geneious Pro 5.0 (Biomatters, LTD, Auckland, NZ). Unique COI haplotypes and nuclear genotypes were identified using the Haplotype Collapser and Converter option in FaBox 1.35 (http://birc.au.dk/fabox) and deposited in GenBank [accession numbers: KC505629-30, 18S; KC517382-KC517405, COI; because GenBank only accepts sequences ≥200 bp we have included a list of the alleles for the ATPSβ intron in Supporting Information (Text S2)]. After trimming, the allelic state of all 18S sequences were unambiguous with only *Camallanus cotti* sequence (EF180071) having a single heterozygous site. Allelic states of the ATPSβ sequences with more than one heterozygous site (5 sequences) were estimated using the Bayesian program PHASE 2.1 [32], [33] as implemented in the program DnaSP 5.0 [34]. We conducted three runs each for 10,000 iterations with 1000 burn-in iterations and with a unique random-number seed. All runs returned consistent allele identities.

### Phylogenetic analyses

To determine the evolutionary relationship among *S. istiblenni* populations, an intra-specific phylogeny was produced for each locus using maximum likelihood (ML) methods and default settings in the program MEGA 5 [35]. Trees were rooted with sequences obtained from Genbank (18S: *Dracunculus insignis*, AY947719; *D. medinensis*, AY947720; ATPSβ: *Caenorhabditis elegans*, AL023815; COI: *Camallanus cotti*, EU598817-19). For comparison, 18S sequences from the family Camallanidae of Nadler et al. (6) (*Spirocamallanus monotaxis*, JF803931; *S. rebecae*, DQ442667; *S. pintoi*, DQ442666; *S. pacificus*, DQ442665; *Camallanus cotti*, EF180071; *C. lacustris*, DQ442663; *C. oxycephalus*, DQ503463, *C.* sp., DQ442664) were included in the analysis. Bootstrap support values were calculated using default settings with 1000 replicates. The ML tree topology was confirmed using Bayesian Markov Chain Monte Carlo (MCMC) analysis as implemented in mrbayes 3.1.1 [36]. The Bayesian analysis was run using the recommended GTR model with gamma distributed rate variation across sites and a proportion of invariable sites. Simulations were run for one million generations with a sample frequency of 10 and a burn-in of 2500 generations.

Average percent divergence (*d*) between lineages was calculated in arlequin 3.5 [37] using 20,000 permutations (corrected values reported).

### Population genetics: cytochrome oxidase I

Population genetic analyses were conducted to determine the level of similarity between French Polynesia (Marquesas and Society Islands) and Hawai'i populations. Summary statistics, including haplotype diversity (*h*) and nucleotide diversity (π), were estimated with algorithms from Nei [38] as implemented in arlequin (Table 2). To examine the relationships between mitochondrial haplotypes, a phylogenetic median-joining network was constructed using network 4.5 with default settings [39]. Analyses of molecular variance (AMOVA) were performed in arlequin using 20,000 permutations. Wright's *F_ST_* was calculated to detect significant haplotype frequency shifts and was not used to measure conventional population structure or to make estimates of migration. To compare genetic diversity in the introduced and source populations, while controlling for unequal sample sizes, we estimated haplotype richness using rarefaction analysis (analytic rarefactation 1.4; UGA Stratigraphy Lab website; http://www.uga.edu/~strata/software/).

Differences between the proportion of infected fish (prevalence) in Hawai'i versus in French Polynesia were tested using a chi-square (**χ^2^**) goodness-of-fit test [40]. Because *L. kasmira* first acquire *S. istiblenni* as juveniles, one might expect older and larger fish to be more likely to harbor parasites. To ensure that differences in prevalence were not due to sampling bias, we also used an ANCOVA to test for the effect of fish size (weight in grams was chosen because we had a complete dataset) on the proportion of fish infected. We tested for differences between the number of parasites per infected fish (intensity) using an unpaired t-test. To control for fish size, we also tested for differences in the number of parasites per gram of infected fish. Calculations were conducted using the online calculator GraphPad Prism (http://www.graphpad.com/prism/statistics.htm).

## Results

Fish sampled in Hawai'i were significantly larger than those sampled in French Polynesia (Hawai'i: N = 288, mean fish weight = 190.1 g, SE = 6.07; French Polynesia: N = 300, mean fish weight = 137.6 g, SE = 4.92; unpaired t-test, t = 6.75, *P*<0.001). At the archipelago level, 34.7% of *L. kasmira* sampled in French Polynesia were infected (Table 1). In Hawai'i this number was higher with 63.5% of *L. kasmira* infected (χ^2^ = 37.2, *P*<0.001) and when just the southeastern half of the archipelago (the region of introduction) was considered (Fig. 2, HI to FF), the infection rate was even higher, with 82.3% of *L. kasmira* infected. Although fish size was significantly related to infection rate (ANCOVA, F = 9.9, *P* = 0.002), there was also a significant effect of geographic location (Hawai'i vs. French Polynesia) after controlling for the effect of fish size (ANCOVA, F = 37.8, *P*<0.001). Hawaiian *L. kasmira* also harbored more parasites per infected host than populations in their native range of French Polynesia (Hawai'i: N = 181, mean number per infected fish = 6.9, SE = 0.4; French Polynesia: N = 105, mean number per infected fish = 2.6, SE = 0.2; unpaired t-test, t = 2.74, *P* = 0.044). This relationship was still significant after correcting for fish size (Hawai'i: N = 181, mean number per g^−1^ = 0.045, SE = 0.003; French Polynesia: N = 105, mean number per g^−1^ = 0.019, SE = 0.001; unpaired t-test, t = 5.62, *P*<0.001). The parasite was absent from most locations northwest of French Frigate Shoals in Hawai'i, with only a small proportion of individuals infected (2 of 13 individuals at Pearl and Hermes Atoll; Fig. 2).

Our sampling effort indicates that *S. istiblenni* is either absent or rare in other regions of the South Pacific. We found no spirocamallanids in 7 *L. kasmira* from the Northern Line Islands, the closest island group south of Hawai'i. Only 4.5% of 199 *Cephalopholis argus* (mean intensity = 1.8 parasites per infected fish) and none of the 131 *Lutjanus fulvus* sampled in the Northern Line Islands were infected with spirocamallanids. We detected 3 spirocamallanids in 40 *L. kasmira* from Fiji (prevalence = 7.5%, mean intensity = 1.3) and no spirocamallanids in 14 *L. kasmira* from American Samoa.

### Phylogenetic analyses

**Figure 3:**
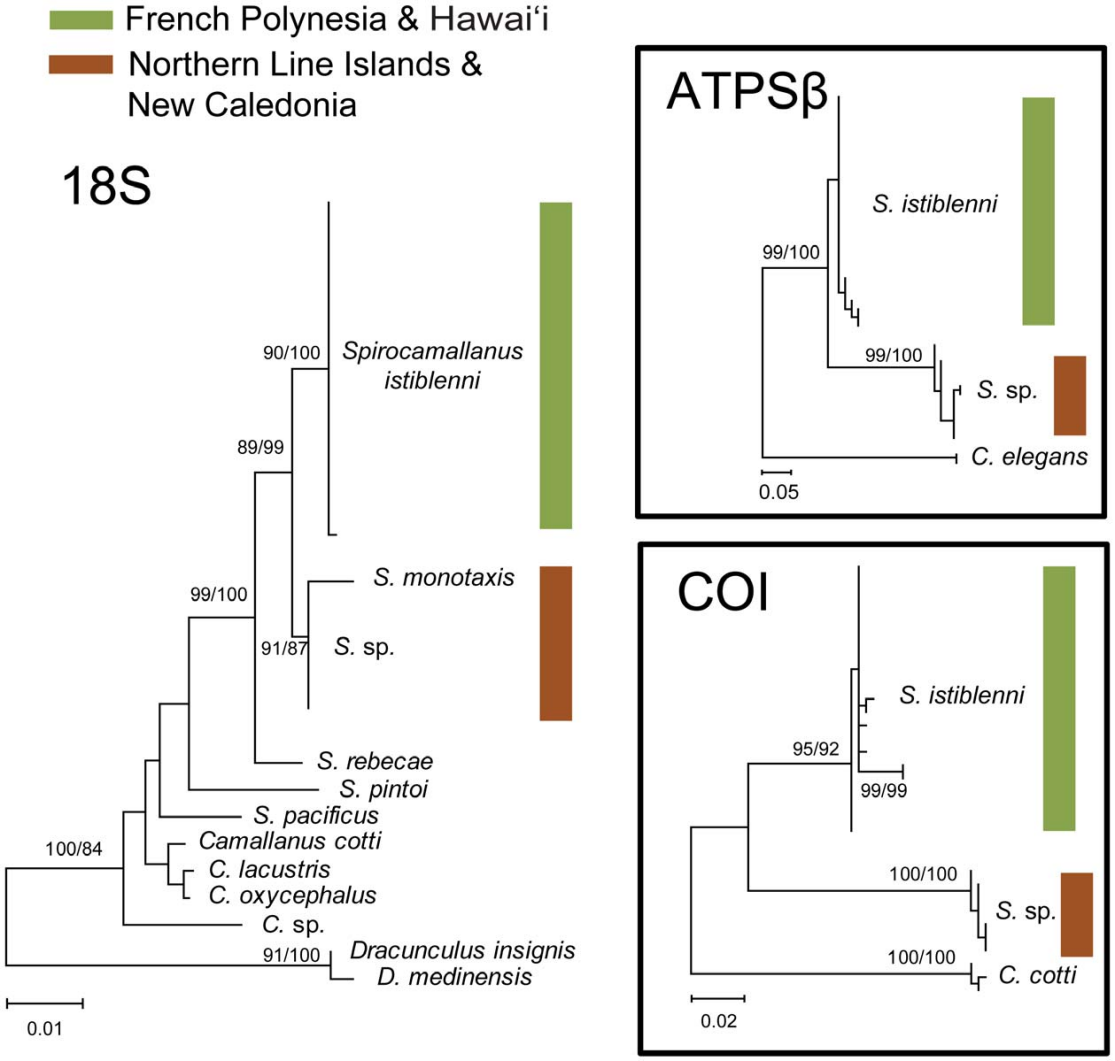
Phylogenetic trees for 18S and ATPSβ alleles and COI haplotypes from *Spirocamallanus istiblenni*. The best maximum likelihood tree generated using program default settings in mega 5 [31]. Bootstrap support values were calculated using default settings with 1000 replicates. For comparison Bayesian posterior probabilities are presented. Colored bars delineate the two divergent lineages detected in French Polynesia and Hawai'i (green) in the Northern Line Islands (brown).

We resolved 1039 bp of 18S rDNA in 30 parasites (Hawai'i = 9, Marquesas = 9, Society = 7, Line Islands = 5) resulting in two alleles, 92 bp of the ATPSβ intron in 31 parasites (Hawai'i = 8, Marquesas = 8, Society = 8, Line Islands = 7) resulting in eight alleles and 362 bp of mitochondrial COI in 30 parasites (Hawai'i = 8, Marquesas = 8, Society = 7, Line Islands = 7) resulting in 10 haplotypes (Table S2). These loci reveal two well-supported and divergent lineages within the spirocamallanids sampled here (18S, 0.7%; ATPSβ, 19.1%; COI, 11.2%; Fig. 3). *S. istiblenni* from French Polynesia and Hawai'i form one lineage while a second lineage consists of *S. monotaxis* and an unidentified *Spirocamallanus* sp. collected in the Northern Line Islands. For comparison we obtained sequences of camallanids from GenBank (see Methods). The 18S tree shows that species-level divergences within the family Camallanidae range from 0.2% (*Camallanus lacustris* vs. *C. oxycephalus*) to 3.7% (*Spirocamallanus monotaxis* vs. *S. pintoi*), indicating that the 0.7% divergence between the two lineages (French Polynesia and Hawai'i versus Northern Line Islands) likely represents species-level divergence. Corroborating this finding is a high level of divergence detected between the two lineages in both the ATPSβ intron (19.1%) and the mitochondrial COI (11.2%) (no corresponding *S. monotaxis* sequences were available for these markers). The phylogenetic grouping of our Northern Line Islands spirocamallanids in the 18S tree indicates that these specimens could be *S. monotaxis*, a closely related nematode that is morphologically differentiated only by the relative position of anal papillae [22].

### Population genetics

**Figure 4:**
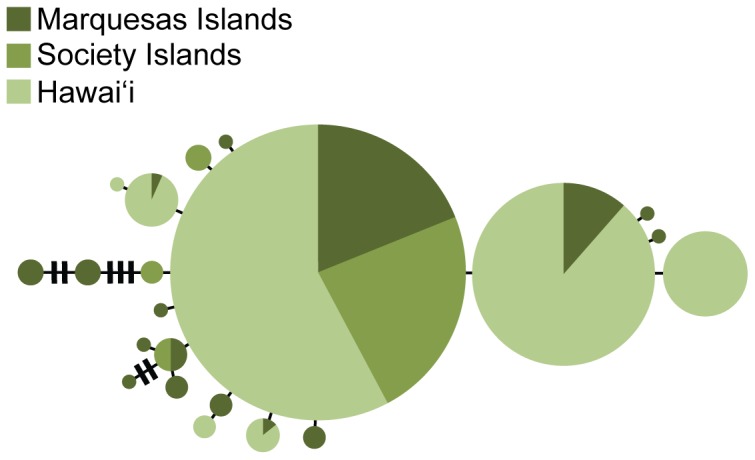
Median Joining network for 383 COI sequences of *Spirocamallanus istiblenni*. Only specimens collected from French Polynesian (Marquesas and Society Islands) and Hawai'i are shown. Each circle represents one mitochondrial haplotype with the area of each circle proportional to number of that particular haplotype in the data set; colors represent sampling location (see key).

We resolved a 362 bp segment of COI in 383 *S. istiblenni* yielding 21 haplotypes with 7 of these observed in single individuals (Table 3; Fig. 4). Based on COI sequences, the combined source populations harbored greater genetic diversity (haplotypes: Marquesas = 16, Society Islands = 4) than the introduced population (Hawai'i = 7; Table 2, Fig. 4). The most common haplotype in French Polynesia was detected at each sample location in Hawai'i (Table 3) including parasites collected from the native fish *Monotaxis grandoculis* (M.R.G. unpublished data).

**Table 3:** Haplotype frequencies for the cytochrome oxidase I gene (COI) for *Spirocamallanus istiblenni*.

	French Polynesia		Hawai'i							
Haplotype	MI	SI	HI	MA	OA	NI	NE	FF	PH	Total
Sis1	34	42	17	14	18	25	2	20	3	175
Sis2		3								3
Sis3		2								2
Sis4	14		9	19	25	19		23	2	111
Sis5	2									2
Sis6	2	2								4
Sis7	3									3
Sis8	1									1
Sis9	2									2
Sis10	2									2
Sis11	3									3
Sis12	1		1	4		2		6		14
Sis13			3	5	2	6		6	1	23
Sis14				2						2
Sis15	1									1
Sis16	1									1
Sis17	1									1
Sis18	1									1
Sis19	1		1	1		3				6
Sis20	1									1
Sis21					1					1
Total	70	49	31	45	46	55	2	55	6	383

Specimens were collected from the host fish *Lutjanus kasmira*. See Table 1 for abbreviations.

**Figure 5:**
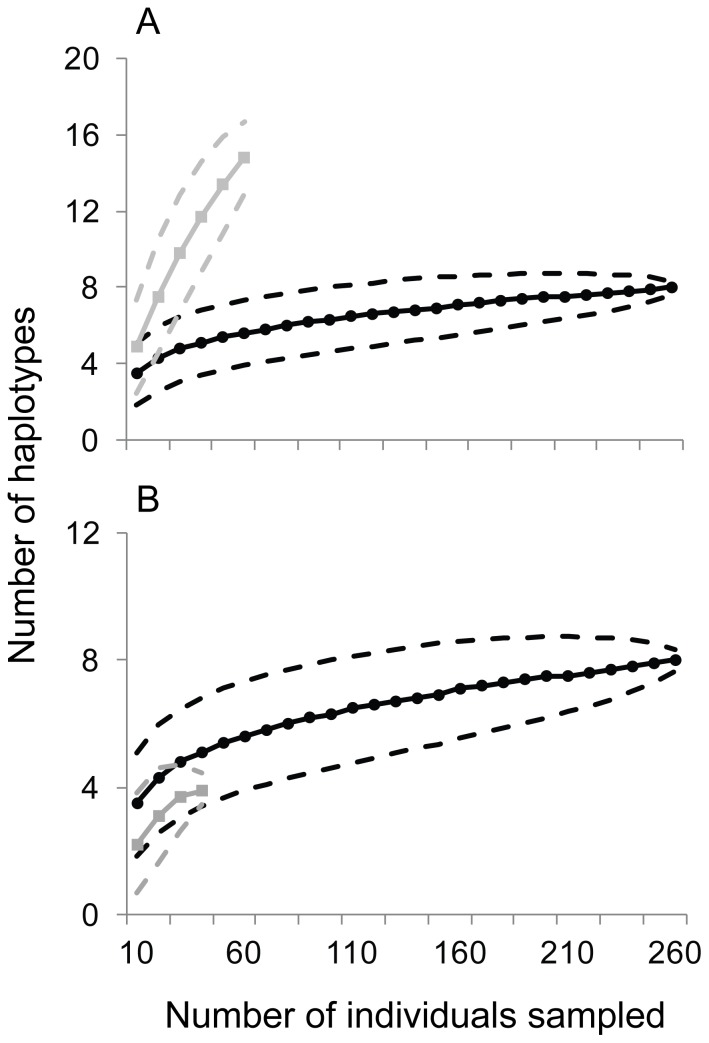
Rarefaction curves plotting the number of individuals sampled against the expected number of mitochondrial haplotypes. Samples from the source populations at the Marquesas (a) and Society (b) Islands are plotted separately. Grey lines represent data from source populations, black lines represent data from Hawai'i, and dashed lines are 95% confidence intervals.

Rarefaction analysis indicates that there was no significant difference in the number of expected mtDNA haplotypes in Hawai'i compared to the source population in the Society Islands (Fig. 5). However, Hawaiian populations harbor significantly less mtDNA diversity than the other source population in the Marquesas (Fig. 5). We found no evidence of haplotype frequency shifts among the islands in the introduced range with overall *F_ST_* in Hawai'i = −0.008 (*P* = 0.446).

## Discussion

Here, we combine molecular, historical, and ecological data to resolve the cryptogenic status of the parasitic nematode *Spirocamallanus istiblenni* in Hawai'i. Phylogenetic analyses reveal a lineage of *S. istiblenni* in French Polynesia that was also detected in Hawaiian specimens of the introduced fish *L. kasmira*. Despite the 3,500 km that separate French Polynesia and Hawai'i, all *S. istiblenni* collected from Hawai'i nested within the French Polynesian lineage (Fig. 3). Indeed, *S. istiblenni* at these geographically distant locations shared most COI haplotypes, with the Hawaiian samples representing a subset of the more diverse French Polynesian haplotypes (Table 3, Fig. 4). Despite intense sampling efforts in the Northern Line Islands (Table 1), which is geographically intermediate between French Polynesia and Hawai'i (and a predominant route of natural colonization into Hawai'i), we found only 9 spirocamallanids in this island group, and all were genetically distinct from the French Polynesian/Hawaiian lineage (18S, *d* = 0.7%), grouping with *S. monotaxis* in our phylogenetic tree: an unlikely scenario if *S. istiblenni* had colonized Hawai'i via natural dispersal.

*S. istiblenni* infects 82% of the *L. kasmira* in the Main Hawaiian Islands where the original introductions took place. While the host fish spread rapidly throughout the archipelago, reaching the far western Midway Atoll within 34 years [16], [41], the parasite has lagged behind and is only prevalent as far as French Frigate Shoals, about half-way up the island chain (Fig. 2). Only two infected fish have been found in the northwest end of the archipelago, indicating that the range of this alien parasite is still expanding, and like that of its host *L. kasmira* [42], may eventually span the entire archipelago. An abundance of host fish species in the northwest Hawaiian Islands, and at least two native copepods that can act as intermediate hosts for *S. istiblenni* (G.A. unpublished data), both support this hypothesis.

### Genetic evidence: Hawaiian populations show founder effect

The Hawaiian population of *S. istiblenni* is dominated by two haplotypes (Table 3, Fig. 4) that constitute 79% of the genetic diversity. Only 7 haplotypes were detected in Hawai'i compared to 18 in French Polynesia (Table 2), and rarefaction analyses confirmed the loss of genetic diversity during the initial introduction (Fig. 5); a pattern expected but often not detected in introduced species [43] including parasites [44] (but see also [45]). We found no evidence of founder events (i.e. shifts in haplotype frequencies) as *S. istiblenni* subsequently colonized the Hawaiian Archipelago (overall *F_ST_* = −0.008, *P* = 0.446). This finding is similar to the host fish, *L. kasmira*, in which high genetic diversity and no population structure was detected within the introduced range, indicating that the host and parasite colonized each island in sufficient numbers to capture most of the standing genetic diversity [16], [42].

### Ecological evidence: parasite lags behind host in introduced range

A lag between host and parasite geographic distributions during range expansion has been observed in other systems [46] and is suspected to result from either founder effects or lowered transmission rates at the invasion front due to density-dependent processes [47]. However, our case requires an alternate explanation because host fish colonizing new islands do not carry the parasite. Snappers, and other reef fishes, disperse over long distances as pelagic larvae, and the parasite communities that infect larval fishes generally do not correspond to those of the adults [48], [49]. Instead, range expansion in the parasite is likely mediated by an obligate intermediate host, either a copepod or amphipod, which is required for completion of the parasite life cycle [50], [51]. Therefore, the spread of this parasite is mediated by at least two species whose population dynamics and susceptibility vary independently, which may slow the spread of the parasite relative to its alien host [46], [52]. Evidence that parasite range expansion can lag behind their host has been documented in lugworms from invasive cane toads in Australia [46], but counterexamples include trematodes that infect Japanese marine mud snails in the Eastern Pacific [44]. The latter taxa requires up to three intermediate hosts to complete its lifecycle, while the former is directly transmitted between hosts. The abundance of possible intermediate hosts of *S. istiblenni* (copepods or amphipods), including two species of calanoid copepods (genera *Labidocera* and *Undinula*) that act as intermediate hosts for *S. istiblenni* in laboratory experiments (G.A. unpublished data), indicate that factors other than lifecycle may be slowing the spread of this parasite.

### Competitive release or ecological factors drive prevalence in introduced range

Generally, introduced species harbor lower parasite diversity and suffer lower rates of infection than do conspecifics in their native range [46]. This has been demonstrated in introduced marine fishes in Hawai'i, including *L. kasmira* [14], [53]. Vignon et al. [18] record a loss of at least 13 parasite taxa and an acquisition of only two parasites following the introduction of *L. kasmira* to Hawai'i. For those parasites found to occur in both the native and introduced ranges, prevalence was generally lower in Hawai'i [18]. *S. istiblenni* is an exception. Nearly twice as many *L. kasmira* in Hawai'i are parasitized by *S. istiblenni* compared to the native range and infected fish have an average of 1.9 times more parasites per host, a finding that is again similar to the trematodes that infect Japanese marine mud snails in the Eastern Pacific [44]. The increased prevalence and intensity of *S. istiblenni* in Hawai'i raise the possibility that this parasite benefits from the loss of competing gut parasites [54]–[56]. Alternatively, the increase in prevalence of *S. istiblenni* may reflect favorable habitat in a host fish experiencing reduced stress from both competitors and parasites. Finally, favorable extrinsic factors such as large host populations (including intermediate and definitive hosts) or the presence of alternative hosts not found in the native range, could enhance transmission and lead to increased infection rates in the introduced range. None of these scenarios are mutually exclusive and all could be working in conjunction to result in increased prevalence and intensity of *S. istiblenni* in the introduced range.

### Conclusion

Here, we synthesize phylogeography, population genetics, and ecological survey data to confirm the alien status of the cryptogenic parasitic nematode *S. istiblenni* in Hawai'i. This species was brought to Hawai'i over 50 years ago during well-intentioned efforts to enhance local fisheries. The outcome for fisheries has been just the opposite, with *L. kasmira* regarded as a nuisance and, by some, as a threat to native species. Here we show that the introduced fish also brought an unwelcome passenger, *S. istiblenni*. Once introduced, *S. istiblenni* attained a two-fold increase in prevalence, as well as a similar increase in intensity. *S. istiblenni* lags behind its primary host *L. kasmira* in the introduced range but there is no obvious barrier to colonization of the entire Hawaiian Archipelago. The threat of exotic parasites to native populations has been well documented and there are several cases of host populations suffering severe consequences due to the impact of alien parasites and disease agents [57]–[60]. This introduced parasite has spread to endemic and other native Hawaiian fishes, and into the protected Papahānaumokuākea Marine National Monument, with consequences that have yet to unfold.

## Supporting Information

Table S1 **List of** ***Spirocamallanus istiblenni*** **specimens deposited in the National Museum of Natural History (MNHN, Paris, France).**
(XLS)

Table S2 **Allele frequencies for 18S rDNA and the ATPSβ intron and haplotypes for COI for** ***Spirocamallanus istiblenni*** **used to reconstruct phylogenies (** **Figure 3** **).**
(DOC)

Text S1 **Species of native Hawaiian fish known to act as hosts for** ***Spirocamallanus istiblenni*** **.**
(DOC)

Text S2 **Alleles of the ATPSβ intron detected in** ***Spirocamallanus istiblenni*** **.**
(DOC)

## References

[1] Cowie RH (1998) Patterns of introduction of non-indigenous non-marine snails and slugs in the Hawaiian Islands. Biodiv Conserv 7: 349–368.

[2] Mack RN, Simberloff D, Lonsdale WM, Evans H, Clout M, et al (2000) Biotic invasions: causes, epidemiology, global consequences, and control. Ecol Appl 10: 689–710.

[3] Pimentel D, Lach L, Zuniga R, Morrison D (2000) Environmental and economic costs of nonindigenous species in the United States. BioSci 50: 53–65.

[4] Pimentel D, Zuniga R, Morrison D (2005) Update on the environmental and economic costs associated with alien–invasive species in the United States. Ecol Econ 52: 273–288.

[5] Tatem AJ, Hay SI, Rogers DJ (2006) Global traffic and disease vector dispersal. Proc Nat Acad Sci 103: 6242–6247. 16606847 10.1073/pnas.0508391103 PMC1435368

[6] Willamson M, Fitter A (1996) The varying success of invaders. Ecology 77: 1661–666.

[7] Cox GW (1999) Alien species in North America and Hawai'i: impacts on natural ecosystems. Washington, DC: Island Press. 387 p.

[8] Simberloff D (2005) Non–native species do threaten the natural environment. J Agri Env Ethics 18: 595–607.

[9] Perrings C, Dehnen–Schmutz K, Touza J, Williamson M (2005) How to manage biological invasions under globalization. Trends Ecol Evol 20: 212–215. 16701371 10.1016/j.tree.2005.02.011

[10] Schlaepfer MA, Sherman PW, Blossey B, Runge MC (2005) Introduced species as evolutionary traps. Ecol Lett 8: 241–246.

[11] Godwin S, Rodgers KS, Jokiel PL (2006) Reducing potential impact of invasive marine species in the Northwestern Hawaiian Islands marine national monument. Honolulu: Northwest Hawaiian Islands Marine National Monument Administration. 66 p.

[12] Carlton JT (1996) Biological invasions and cryptogenic species. Ecology 77: 1653–1655.

[13] Concepcion GT, Kahng SE, Crepeau MW, Franklin EC, Coles SL, et al (2010) Resolving natural ranges and marine invasions in a globally distributed octocoral (genus *Carijoa*). Mar Eco Prog Ser 401: 113–127.

[14] Vignon M, Sasal P (2010) Fish introduction and parasites in marine ecosystems: a need for information. Environ Biol Fish 87: 1–8.

[15] Schumacher BD, Parrish JD (2005) Spatial relationships between an introduced snapper and native goatfishes on Hawaiian reefs. Biol Inv 7: 925–933.

[16] Gaither MR, Bowen BW, Toonen RJ, Planes S, Messmer V, et al (2010) Genetic consequences of introducing two allopatric lineages of Bluestripe Snapper (*Lutjanus kasmira*) to Hawai'i. Mol Ecol 19: 1107–1121. 20163550 10.1111/j.1365-294X.2010.04535.x

[17] Randall JE, Kanayama RK (1982) Hawaiian fish immigrants. Sea Front 18: 144–15.

[18] Vignon M, Sasal P, Rigby MC, Galzin R (2009) Multiple parasite introduction and host management plan: case study of lutjanid fish in the Hawaiian Archipelago. Dis Aquat Org 85: 133–145. 19694173 10.3354/dao02071

[19] Font WF, Rigby M (2000) Implications of a new Hawaiian host record from blue–lined snappers *Lutjanus kasmira*: Is the nematode *Spirocamallanus istiblenni* native or introduced? Bishop Mus Occas Pap 64: 53–56.

[20] Meguid MA, Eure HE (1996) Pathobiology associated with the spiruroid nematodes *Camallanus oxycephalus* and *Spinitectus carolini* in the intestine of green sunfish, *Lepomis cyanellus* . J Parasitol 82: 118–123. 8627480

[21] Menzes RC, Tortelly R, Tortelly–Neto R, Noronha D, Pinto RM (2006) *Camallanus cotti* Fujita, 1927 (Nematoda, Camallanoidea) in ornamental aquarium fishes: pathology and morphology. Mem Inst Oswaldo Cruz 101: 683–687. 17072484 10.1590/s0074-02762006000600018

[22] Rigby MC, Font WF (2001) Statistical reanalysis of the distinction between *Spirocamallanus istiblenni* and *S. monotaxis* (Nematoda: Camallanidae). J Parasitol 87: 1213–1215. 11695404 10.1645/0022-3395(2001)087[1210:SROTDB]2.0.CO;2

[23] Rigby MC, Font WF (1997) Redescription and range extension of *Spirocamallanus istiblenni* Noble, 1966 (Nematoda: Camallanidae) from coral reef fishes in the Pacific. J Helminthol Soc Wash 64: 227–233.

[24] Randall JE (1998) Zoogeography of shore fishes of the Indo-Pacific region. Zool Stud 37: 227–268.

[25] Skillings DJ, Bird CE, Toonen RJ (2011) Gateways to Hawai'i: genetic population structure of the tropical sea cucumber *Holothuria atra* . J Mar Biol 2011: Article ID 783030 doi:10.1155/2011/783030.

[26] Seutin G, White BN, Boag PT (1991) Preservation of avian and blood tissue samples for DNA analyses. Can J Zool 69: 82–92.

[27] Truett GE, Mynatt RL, Truett AA, Walker JA, Warman ML (2000) Preparation of PCR-quality mouse genomic DNA with hot sodium hydroxide and Tris (HotSHOT). Bio Tech 29: 52–54. 10.2144/00291bm09 10907076

[28] Meeker ND, Hutchinson SA, Ho L, Trede NS (2007) Method for isolation of PCR-ready genomic DNA from zebrafish tissues. BioTech 43: 610–614. 10.2144/000112619 18072590

[29] Wu SG, Wang GT, Xi BW, Xiong F, Liu T, et al (2009) Population genetic structure of the parasitic nematode *Camallanus cotti* inferred from DNA sequences of ITS1 rDNA and the mitochondrial COI gene. Veter Parasitol 164: 248–256. 10.1016/j.vetpar.2009.04.030 19632785

[30] Nadler S (2007) Molecular phylogeny of clade III nematodes reveals multiple origins of tissue parasitism. Parasitol 134: 1421–1442. 10.1017/S0031182007002880 17506928

[31] Jarman SN, Ward RD, Elliott NG (2002) Oligonucleotide primers for PCR amplification of coelomate introns. Mar Biotech 4: 347–355. 10.1007/s10126-002-0029-6 14961246

[32] Stephens M, Smith NJ, Donnelly P (2001) A new statistical method for haplotype reconstruction from population data. Am J Hum Genet 68: 978–989. 11254454 10.1086/319501 PMC1275651

[33] Stephens M, Donnelly P (2003) A comparison of Bayesian methods for haplotype reconstruction from population genotype data. Am J Hum Genet 73: 1162–1169. 14574645 10.1086/379378 PMC1180495

[34] Librado P, Rozas J (2009) DnaSP v5: As software for comprehensive analysis of DNA polymorphism data. Bioinform 25: 1451–1452. 10.1093/bioinformatics/btp187 19346325

[35] Tamura K, Dudley J, Nei M, Kamar S (2007) MEGA4: molecular evolutionary genetics analysis (MEGA) software version 4.0. Mol Biol Evol 24: 1596–1599. 17488738 10.1093/molbev/msm092

[36] Huelsenbeck JP, Ronquist F (2001) MRBAYES: Bayesian inference of phylogeny. Bioinform 17: 754–755. 10.1093/bioinformatics/17.8.754 11524383

[37] Excoffier L, Lischer HEL (2010) Arlequin suite ver 3.5: a new series of programs to perform population genetics analyses under Linux and Windows. Mol Ecol Res 10: 564–567. 10.1111/j.1755-0998.2010.02847.x 21565059

[38] Nei M (1987) Molecular evolutionary genetics. New York: Columbia University Press. 512 p.

[39] Bandelt HJ, Forster P, Röhl A (1999) Median–joining networks for inferring intraspecific phylogenies. Mol Biol Evol 16: 37–48. 10331250 10.1093/oxfordjournals.molbev.a026036

[40] Sokal RR, Rohlf JF (1995) Biometry: the principles and practice of statistics in biological research. New York: W.H. Freeman and Company. 887 p.

[41] Randall JE (1987) Introductions of marine fishes to the Hawaiian Islands. Bull Mar Sci 47: 356–400.

[42] Gaither MR, Toonen RJ, Bowen BW (2012) Coming out of the starting blocks: Extended lag time rearranges genetic diversity in introduced marine fishes of Hawai'i. Proc Roy Soc B 279: 3948–3957 doi:10.1098/rspb.2012.1481. 10.1098/rspb.2012.1481 PMC3427594 22874747

[43] Roman J, Darling JA (2007) Paradox lost: genetic diversity and the success of aquatic invasions. Trends Ecol Evol 22: 454–464. 17673331 10.1016/j.tree.2007.07.002

[44] Miura O, Torchin ME, Kuris AM, Hechinger RF, Chiba S (2006) Introduced cryptic speices of parasites exhibit different invasion pathways. PNAS 103: 19818–19823. 17179044 10.1073/pnas.0609603103 PMC1750888

[45] Stefani F, Aquaro G, Azzurro E, Colorni A, Galli P (2012) Patterns of genetic variation of a Lessepsian parasite. Biol Invasions 14: 1725–1736.

[46] Torchin ME, Lafferty KD, Dobson AP, McKenzie VJ, Kuris AM (2003) Introduced species and their missing parasites. Nature 421: 628–630. 12571595 10.1038/nature01346

[47] Phillips BL, Kelehear C, Pizzatto L, Brown GP, Barton D, et al (2010) Parasites and pathogens lag behind their host during periods of host range advance. Ecology 91: 872–881. 20426344 10.1890/09-0530.1

[48] Rigby MC, Dufour V (1996) Parasites of coral reef fish recruits, *Epinephelus merra* (Serranidae), in French Polynesia. J Parasitol 82: 405–408. 8636843

[49] Cribb TH, Pichelin S, Dufour V, Bray RA, Chauvet C, et al (2000) Parasites of recruiting coral reef fish larvae in New Caledonia. Int J Parasitol 30: 1445–1451. 11428334 10.1016/s0020-7519(00)00121-1

[50] Anderson RC (2000) Nematode parasites of vertebrates: their development and transmission. Wallingford: CABI Publishing. 650 p.

[51] Rigby MC (2013) Order Camallanida: Superfamilies Anguillicoloidea and Camallanoidea. In: Schmidt–Rhaesa A, editor. Handbook of Zoology: A Natural History of the Phyla of the Animal Kingdom Gastrotricha, Cycloneuralia, Gnathifer. de Gruyter, Berlin.

[52] Guégan J–F, Kennedy CR (1993) Maximum local helminth parasite community richness in British freshwater fish: a test of the colonization time hypothesis. Parasitol 106: 91–100. 10.1017/s0031182000074862 8479807

[53] Vignon M, Sasal P, Galzin R (2009) Host introduction and parasites: a case study on the parasite community of the peacock grouper *Cephalopholis argus* (Serranidae) in the Hawaiian Islands. Parasitol Res 104: 775–782. 19002714 10.1007/s00436-008-1254-3

[54] Read CP (1951) The “crowding effect” in tapeworm infections. J Parasitol 37: 174–178. 14841561

[55] Holmes JC (1961) Effects of concurrent infections on *Hymenolepis dininuta* (Cestoda) and *Moniliformis dubius* (Acanthocephala). I. general effects and comparisons with crowding. J Parasitol 47: 209–216. 13715464

[56] Bush AO, Lotz JM (2000) The ecology of “crowding.”. J Parasitol 86: 212–213. 10780534 10.1645/0022-3395(2000)086[0212:TEOC]2.0.CO;2

[57] Dobson A, Foufopoulos J (2001) Emerging infectious pathogens of wildlife. Phil Trans R Soc Lond B 356: 1001–1012. 11516378 10.1098/rstb.2001.0900 PMC1088495

[58] Grosholz E (2002) Ecological and evolutionary consequences of coastal invasions. Trends Ecol Evol 18: 22–27.

[59] Prenter J, MacNeil C, Dick JTA, Dunn AM (2004) Roles of parasites in animal invasions. Trends Ecol Evol 19: 385–390. 16701290 10.1016/j.tree.2004.05.002

[60] Bondad–Reantaso MG, Subasinghe RP, Arthur JR, Ogawa K, Chinabut S, et al (2005) Disease and health management in Asian aquaculture. Vet Parasitol 132: 249–272. 16099592 10.1016/j.vetpar.2005.07.005

