# Supplementary material for: An Invasive Fish and the Time-Lagged Spread of Its Parasite across the Hawaiian Archipelago
Source: PLoS One. 2013 Feb 27;8(2):e56940. doi: 10.1371/journal.pone.0056940 (PMC3584140; doi:10.1371/journal.pone.0056940)
Supplement: Table S2 — Allele frequencies for 18S rDNA and the ATPSβ intron and haplotypes for COI for Spirocamallanus istiblenni used to reconstruct phylogenies ( Figure 3 ). (DOC) [file pone.0056940.s002.doc]

**Table S2.** **Allele frequencies for 18S rDNA and the ATPSβ intron and haplotypes for COI for *Spirocamallanus istiblenni* used to reconstruct phylogenies (Figure 3).**

| Allele | Marquesas | Society | Hawaii | Line Islands |
| --- | --- | --- | --- | --- |
| 18S_Sis1 | 18 | 14 | 18 |  |
| 18S_Sis2 |  |  |  | 10 |
|  |  |  |  |  |
| ATPSβ_Sis1 | 15 | 12 | 14 |  |
| ATPSβ_Sis2 |  | 1 | 2 |  |
| ATPSβ_Sis3 | 1 | 2 |  |  |
| ATPSβ_Sis4 |  | 1 |  |  |
| ATPSβ_Sis5 |  |  |  | 3 |
| ATPSβ_Sis6 |  |  |  | 2 |
| ATPSβ_Sis7 |  |  |  | 2 |
| ATPSβ_Sis8 |  |  |  | 7 |
|  |  |  |  |  |
| COI_Sis1 | 3 | 5 | 3 |  |
| CO1_Sis2 |  | 1 |  |  |
| CO1_Sis4 | 1 |  | 5 |  |
| CO1_Sis5 | 1 |  |  |  |
| CO1_Sis6 |  | 1 |  |  |
| CO1_Sis11 | 2 |  |  |  |
| CO1_Sis12 | 1 |  |  |  |
| CO1_Sis22 |  |  |  | 1 |
| CO1_Sis23 |  |  |  | 3 |
| CO1_Sis24 |  |  |  | 3 |

­­See Methods for GenBank accession numbers. Specimens were collected from the host fish *Lutjanus kasmira* except in Line Islands were specimens were obtained from *Cephalopholis argus*.
