# Supplementary material for: An Invasive Fish and the Time-Lagged Spread of Its Parasite across the Hawaiian Archipelago
Source: PLoS One. 2013 Feb 27;8(2):e56940. doi: 10.1371/journal.pone.0056940 (PMC3584140; doi:10.1371/journal.pone.0056940)
Supplement: Text S1 — Species of native Hawaiian fish known to act as hosts for Spirocamallanus istiblenni . (DOC) [file pone.0056940.s003.doc]

Species of native Hawaiian fish known to act as hosts for *Spirocamallanus istiblenni*:

Yellowfin Goatfish, *Mulloidichthys vanicolensis*

Yellowstripe Goatfish, *Mulloidichthys flavolineatus*

Sidespot Goatfish *Parupeneus pleurostigma*

Zebra Blenny, *Istiblennius zebra*

Rockskipper, *Entomacrodus marmoratus*

Hawaiian Sleeper, *Eleotris sandwicensis*

Bigeye Emperior, *Monotaxis grandoculis*
