# Supplementary material for: An Invasive Fish and the Time-Lagged Spread of Its Parasite across the Hawaiian Archipelago
Source: PLoS One. 2013 Feb 27;8(2):e56940. doi: 10.1371/journal.pone.0056940 (PMC3584140; doi:10.1371/journal.pone.0056940)
Supplement: Text S2 — Alleles of the ATPSβ intron detected in Spirocamallanus istiblenni . (DOC) [file pone.0056940.s004.doc]

>ATPSB_Sis1 [organism=Spirocamallanus istiblenni] ATP Synthetase Subunit beta intron

GGTTTTTAATTCTCTTTTCATGCCCTTTCCCACTGAAATATCAGATTTGAAGTTGACATTC

AGTTGATTTTATGAAAGATAGTGGGAATGTT

>ATPSB_Sis2 [organism=Spirocamallanus istiblenni] ATP Synthetase Subunit beta intron

GGTTTTTAATTCTCTTTTCATGCCCTTTCCCACTGAAATATCAGATTTGAAGTTGACATTC

AGTTTATTTTATGAAAGATAGTGGGAATGTT

>ATPSB_Sis3 [organism=Spirocamallanus istiblenni] ATP Synthetase Subunit beta intron

GGTTTTTAATTCTCTTTTCATGCCCTTTCCCAATGAAATATCAGATTTGAAGTTGACATTC

AGTTTAGTTTATGAAAGATAGTGGGAATGTT

>ATPSB_Sis4 [organism=Spirocamallanus istiblenni] ATP Synthetase Subunit beta intron

GGTTTTTAATTCTCTTTTCATGCCCTTTCCCAATGAAATATCAGATTTGAAGTTGACATTC

AGTTTATTTTATGAAAGATAGTGGGAATGTT

>ATPSB_Sis5 [organism=Spirocamallanus istiblenni] ATP Synthetase Subunit beta intron

GGTTTTTAATTCCCTTTTCGCGCATTTTCCCACTACCATATCAGATCTGGAGTCGATATTC

GGTTGAGTTTTGAAAGATATTGGTAATGTT

>ATPSB_Sis6 [organism=Spirocamallanus istiblenni] ATP Synthetase Subunit beta intron

GGTTTTTAATTTCCTTTTCGCGCATTTTTCCACTACCATATCAGATCTGGAGTCGATATTC

GGTTGAGCTTTGAAAGATATTGGTAATGTT

>ATPSB_Sis7 [organism=Spirocamallanus istiblenni] ATP Synthetase Subunit beta intron

GGTTTTTAATTCCCTTTTCGCGCATTTTCCCACTAGCATATCAGATCTGGAGTCGATATTC

GGTTGAGTTTTGAAAGATATTGGTAATGTT

>ATPSB_Sis8 [organism=Spirocamallanus istiblenni] ATP Synthetase Subunit beta intron

GGTTTTTAATTTCCTTTTCGCGCATTTTTCCACTACCATATCAGATCTGGAGTCGATATTC

GGTTGAGTTTTGAAAGATATTGGTAATGTT
